# Control of asymmetric biaryl conformations with terpenol moieties: Syntheses, structures and energetics of new enantiopure *C*_2_-symmetric diols

**DOI:** 10.3762/bjoc.4.25

**Published:** 2008-07-10

**Authors:** Y Alpagut, B Goldfuss, J-M Neudörfl

**Affiliations:** 1 Institut für Organische Chemie, Universität zu Köln, Greinstrasse 4, D-50939 Köln, Germany. Fax: +49(0)221-470-5057, http://www.uni-koeln.de/goldfuss.

**Keywords:** chiral diols, hydrogen bonds, axial chirality, terpenes

## Abstract

New enantiopure, *C*_2_-symmetric biphenyl-2,2′-diols based on (−)-menthone (**BIMOL**), (−)-verbenone (**BIVOL**) and (−)-carvone (**BICOL** and hydrogenated **BIMEOL**), are accessible *via* short, synthetic routes. All diols form intramolecular hydrogen bonds and hence can be employed as chelating ligands for catalyst design, as it demonstrated for the (−)-fenchone based **BIFOL**. The sense of asymmetry of the biphenyl axes is controlled by the chiral terpene units and is conformationally surprisingly stable. X-ray analyses reveal *M* biphenyl conformation for **BIMOL** and *P* biphenyl conformation for each of **BIVOL**, **BICOL** and **BIMEOL**. The origins of the conformational biphenyl preferences are confirmed by computational ONIOM evaluations of the diols and their diastereomeric conformers. The experimentally observed biphenyl conformations are all energetically preferred, i.e. with 1.3 kcal/mol for **(*M*)-BIMOL**, with 5.1 kcal/mol for **(*P*)-BIVOL,** with 5.8 kcal/mol for **(*P*)-BICOL**, and with 5.4 kcal/mol for **(*P*)-BIMEOL.**

## Introduction

**Scheme 1:**
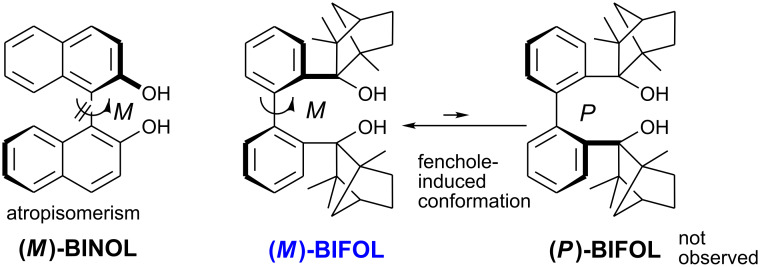
Atropisomeric BINOL and conformationally restricted, (−)-fenchone-based **(*M*)-BIFOL**.

Enantiopure biaryl systems with flexible chiral axes are widespread, e.g. in pharmacological natural products or as ligands in enantioselective catalyses [1]. Chelating *C*_2_-symmetric diols such as BINOLs [2–4] and TADDOLs [5–6] are often employed as chiral ligands in enantioselective synthesis. We recently reported the synthesis and the X-ray crystal structure of **(*M*)-BIFOL** [7] (biphenyl-2,2′-bisfenchol, Scheme 1) and its derivatives [8–11]. **(*M*)-BIFOL** exhibits, in a similar way as BINOLs, a flexible biaryl axis with *M* conformation, induced by the hydrogen bonded fenchol moieties, and sterically crowded aliphatic alcohol functions, as in TADDOLs. The fenchol moieties were shown to stabilize the biphenyl via intramolecular hydrogen bonds [7].

Modular fencholates [12–13] were employed as chiral, chelating ligands, e.g. in enantioselective organozinc catalysts [14–18], in chiral *n*-butyllithium aggregates [19–23] and in enantioselective Pd- and Cu-catalyzed C-C-couplings [9–10,24]. Here we present syntheses and characterizations of new enantiopure *C*_2_-symmetric diols based on (−)-menthone, (−)-verbenone and (−)-carvone, and reveal origins of their conformational biphenyl restrictions.

## Results and Discussion

**Scheme 2:**
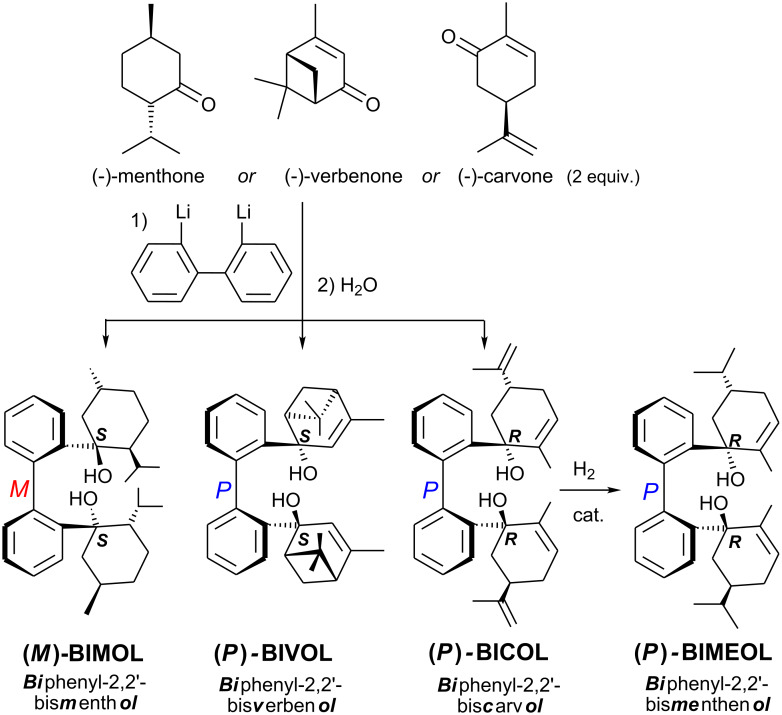
Syntheses of **(*M*)-BIMOL**, **(*P*)-BIVOL**, **(*P*)-BICOL**, and **(*P*)-BIMEOL**.

The new 1,1′-biphenyl-2,2′-bisterpenols **BIMOL**, **BIVOL** and **BICOL** were synthesized by addition of 2,2′-dilithiobiphenyl to (−)-menthone, (−)-verbenone and (−)-carvone and subsequent hydrolysis (Scheme 2). The nucleophilic 2,2′-dilithiobiphenyl adds to the carbonyl groups preferably from the sterically less hindered side, i.e. *trans* to the isopropyl group in (−)-menthone yielding **BIMOL**, and *trans* to the isopropenyl group in (−)-carvone [25–27] yielding **BICOL**. For (−)-verbenone the sterically less crowded side of the pinene backbone, *trans* to the geminal dimethyl unit, is preferred yielding **BIVOL**. Catalytic partial hydrogenation of **BICOL** [28–29] yields the isopropenyl group in **BIMEOL** retaining the cyclohexene unit (Scheme 2).

**Figure 1:**
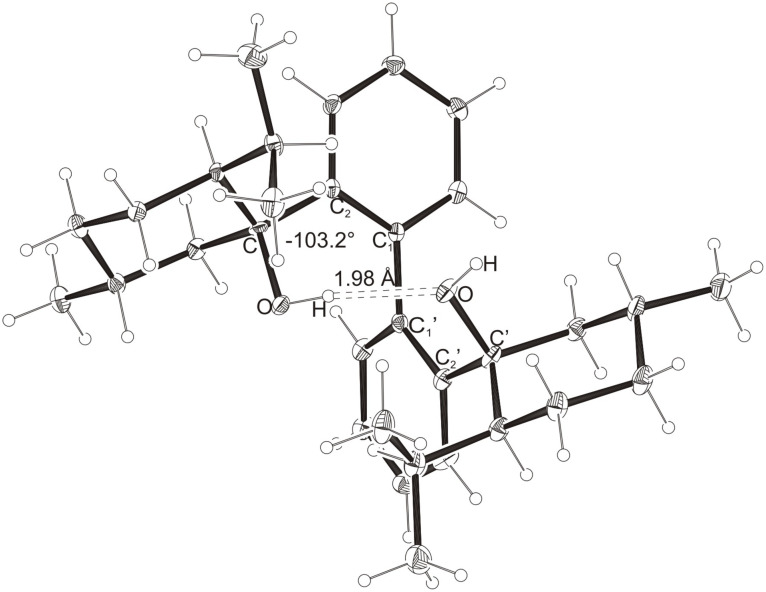
X-Ray crystal structure of **(*M*)-BIMOL**. An acetone molecule, binding to the external OH group, is omitted for clarity.

**Figure 2:**
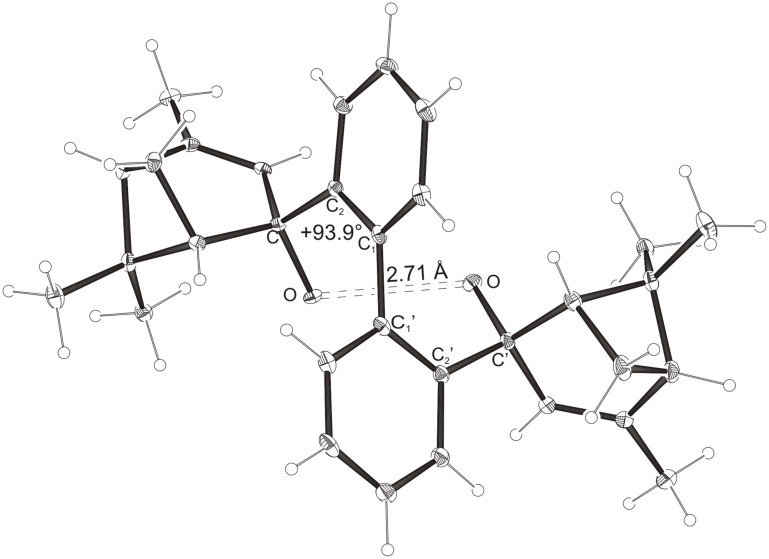
X-Ray crystal structure of **(*P*)-BIVOL**. A water molecule, binding to the external OH group, is omitted for clarity (cf. Scheme 3).

**Figure 3:**
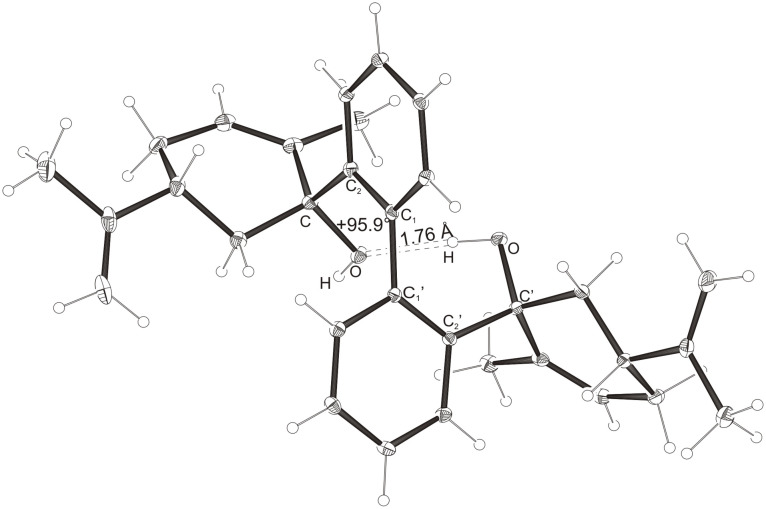
X-Ray crystal structure of **(*P*)-BICOL**. A water molecule, binding to the external OH group, is omitted for clarity (cf. Scheme 3).

**Figure 4:**
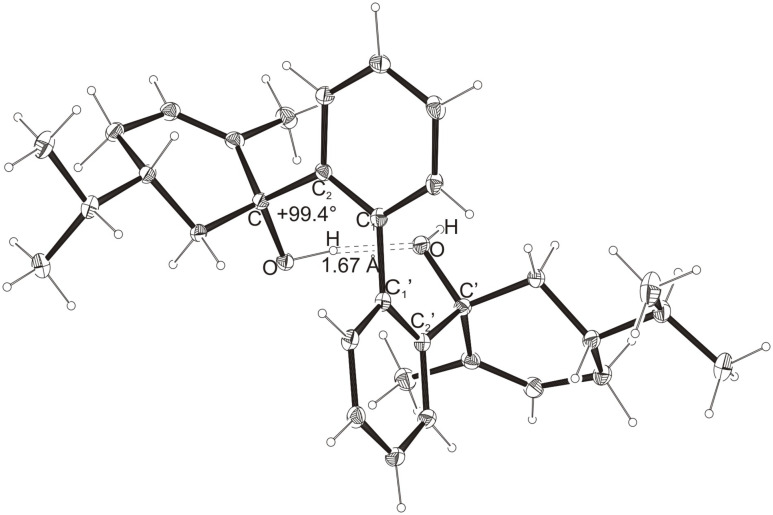
X-Ray crystal structure of **(*P*)-BIMEOL**. An ethanol molecule, binding to the external OH group, is omitted for clarity (cf. Scheme 3)

**Figure 5:**
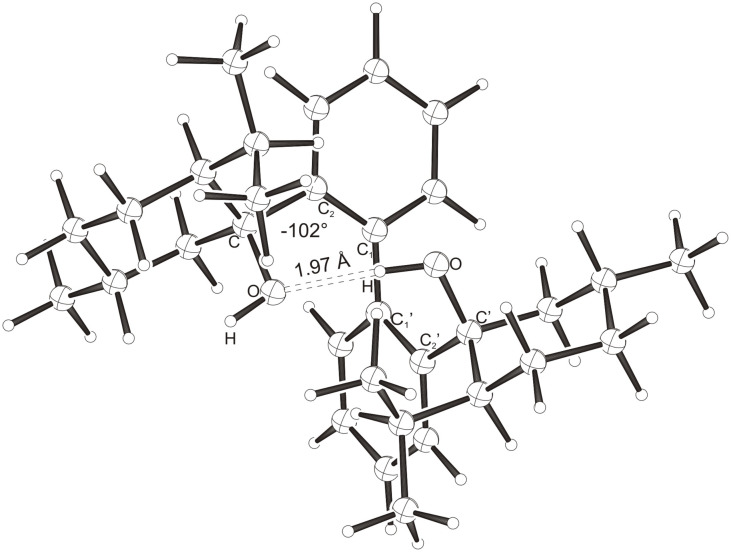
B3LYP/6-31++G**:AM1 optimized structure of **(*M*)-BIMOL**, E_rel._ = 0.0 kcal/mol.

**Scheme 3:**
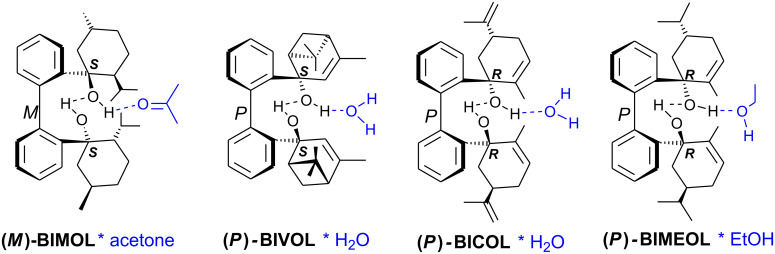
Co-solvent adducts in the X-ray structures of **(*M*)-BIMOL**, **(*P*)-BIVOL**, **(*P*)-BICOL**, and **(*P*)-BIMEOL**.

Intramolecular hydrogen bonds between the hydroxy groups of the terpenol moieties are apparent in the X-ray crystal structures of **BIMOL**, **BIVOL**, **BICOL** and **BIMEOL** (Figure 1 – Figure 4). These chelating hydroxyl groups enable applications in enantioselective catalysts [9,24] and reagents [8], as has been described for the chelating **BIFOL** with 2.2 Å for O-**H-O**H and 3.0 Å for O-O [7]. **BIMOL **exhibits distances of 1.98 Å for O-**H-O**H and of 2.80 Å for O-O (Figure 1, Table 1). In the crystal structure of **BIVOL**, the hydrogen atoms of the hydroxy groups are disordered, but the close distance of 2.71 Å for O-O points also to hydrogen bonding (Figure 2, Table 1). **BICOL** shows distances of 1.76 Å (O-H-OH) and 2.65 Å (O-O, Figure 3, Table 2) and **BIMEOL** of 1.67 Å (O-H-OH) and 2.62 Å (O-O, Figure 4, Table 2). In close analogy to the conformationally restricted **(*M*)-BIFOL** (Scheme 1), all biphenyl axes of these chelating diols exhibit according to X-ray crystal analyses preferred biphenyl conformations: a *M*-(*R*)-sense is found for **BIMOL**, *P*-(*S*)- for **BIVOL**, *P*-(*S)*- for **BICOL** and likewise *P*-(*S*)- for partially hydrogenated **BIMEOL** (Figure 1–Figure 4, Scheme 2). The C2-C1-C1′-C2′ dihedral angles are −103°, +94°, +96° and +99° respectively (Table 1 and Table 2). Computational ONIOM (B3LYP/6-31++G**:AM1) calculations of the chelating diols prove that the experimentally observed biaryl conformations are indeed intrinsically favored, their alternative diastereomeric biphenyl conformers are all energetically disfavored. **(*M*)-BIMOL** is computed to be 1.3 kcal/mol, **(*P*)-BIVOL** is 5.1 kcal/mol**, (*P*)-BICOL** is 5.8 kcal/mol and **(*P*)-BIMEOL** is 5.4 kcal/mol more stable than the conformers **(*P*)-BIMOL**, **(*M*)-BIVOL, (*M*)-BICOL** and **(*M*)-BIMEOL** (Figure 5 – Figure 12, Table 3). The computationally favored and experimentally found conformers can all form hydrogen bonds between the hydroxy groups, due to favorable arrangements of the terpenol units. The disfavored conformers cannot form such close OH-OH contacts, due to repulsion of the unfavorably aligned bulky terpenol moieties. Donor-solvents like acetone, water or ethanol can bind to the external O-H bonds of the diols (Scheme 3), without disrupting the internal O-H-O bridge, which is crucial for the chiral alignment of the biaryl axis.

**Table 1:** Experimental (X-ray crystal structures) and ONIOM(B3LYP/6-31++G**:AM1) computed geometries of **BIMOL** and **BIVOL** in *P* and *M* biphenyl conformations.^a^

	**BIMOL**			**BIVOL**		

	*M*_Xray_	*M*_calc._	*P*_calc._	*P*_Xray_	*P*_calc._	*M*_calc._
O-H-O(H) [ Å ]	1.98	1.973	2.954	-	1.942	2.071
O-O [ Å ]	2.80	2.912	3.678	2.71	2.904	2.957
O-H-O [ ° ]	171.94	160.24	132.32	-	167.31	150.11
C_2_-C_1_-C_1_′-C_2_′ [ ° ]	−103.2	−102.00	+95.21	+93.9	+99.03	−91.61
O-C_Rest_-C_2_-C_1_ [ ° ]	+33.1	+35.55	−4.64	−39.9	−38.16	+108.37
O-C'_Rest_-C_2_′-C_1_′ [ ° ]	+29.6	+28.78	+7.48	−44.7	−42.53	+69.70

^a^The hydroxyl functions were computed by B3LYP, while AM1 was employed for the rest of the structures. Hydrogens were used as link atoms between the layers.

**Table 2:** Experimental (X-ray crystal structures) and ONIOM(B3LYP/6-31++G**:AM1) computed geometries of **BICOL** and **BIMEOL** in *P* and *M* biphenyl conformations.^a^

	**BICOL**			**BIMEOL**		

	*P*_Xray_	*P*_calc._	*M*_calc._	*P*_Xray_	*P*_calc._	*M*_calc._
O-H-O(H) [ Å ]	1.76	2.028	3.666	1.67	2.028	4.055
O-O [ Å ]	2.65	2.967	4.242	2.62	2.966	4.501
O-H-O [ ° ]	169.3	160.15	120.57	168.1	160.02	111.43
C_2_-C_1_-C_1_′-C_2_′ [ ° ]	+95.9	+99.15	−100.26	+99.4	+98.91	−103.47
O-C_Rest_-C_2_-C_1_ [ ° ]	−42.2	−34.01	−28.75	−32.5	−29.27	+1.59
O-C'_Rest_-C_2_′-C_1_′ [ ° ]	−30.1	−29.13	−10.77	−35.6	−34.18	−40.11

^a^The hydroxyl functions were computed by B3LYP, while AM1 was employed for the rest of the structures. Hydrogens were used as link atoms between the layers.

**Figure 6:**
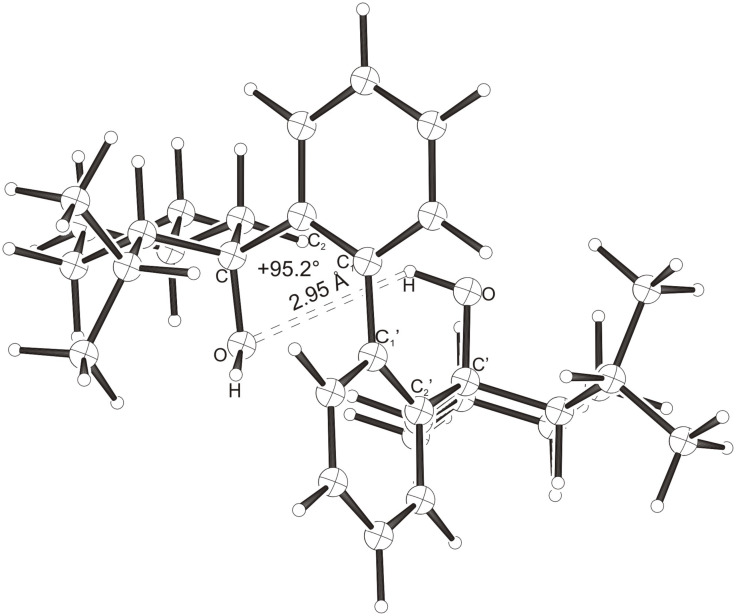
B3LYP/6-31++G**:AM1 optimized structure of **(*P*)-BIMOL**, E_rel._ = 1.3 kcal/mol.

**Figure 7:**
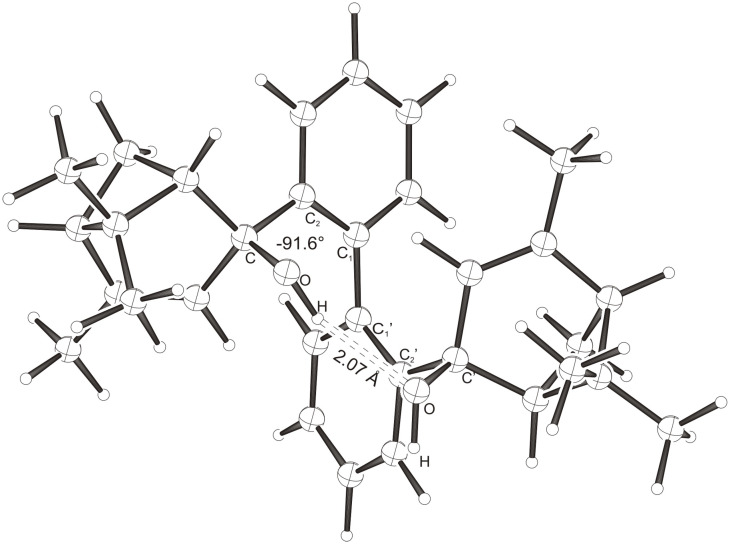
B3LYP/6-31++G**:AM1 optimized structure of **(*M*)-BIVOL**, E_rel._ = 5.1 kcal/mol.

**Figure 8:**
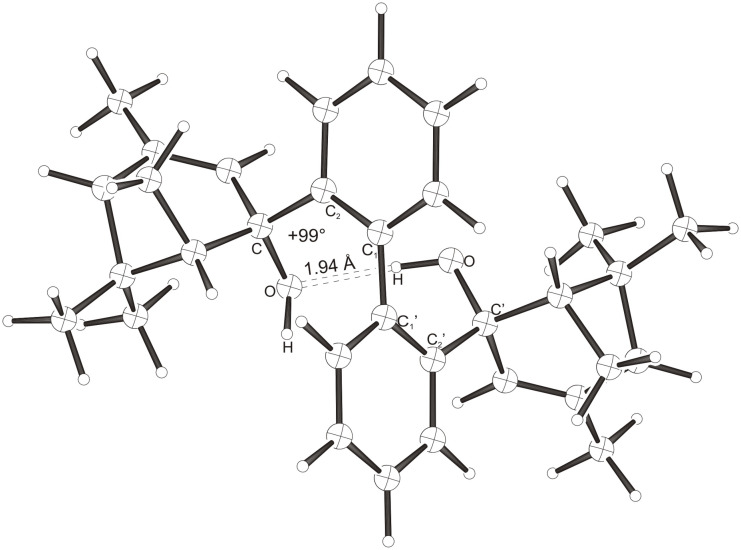
B3LYP/6-31++G**:AM1 optimized structure of **(*P*)-BIVOL**, E_rel._ = 0.0 kcal/mol.

**Figure 9:**
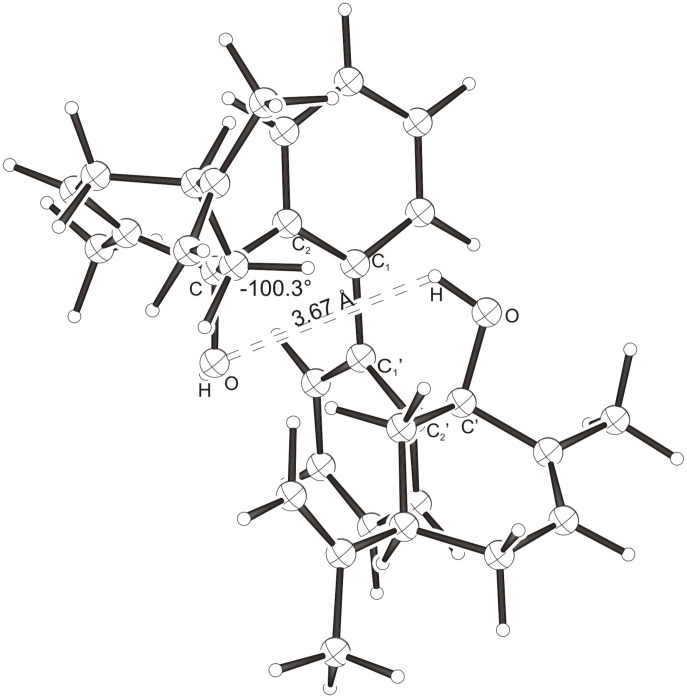
B3LYP/6-31++G**:AM1 optimized structure of **(*M*)-BICOL**, E_rel._ = 5.8 kcal/mol.

**Figure 10:**
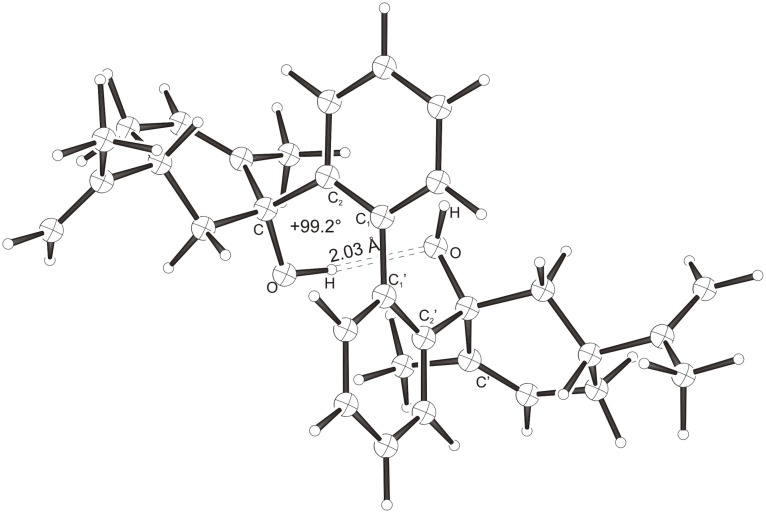
B3LYP/6-31++G**:AM1 optimized structure of **(*P*)-BICOL**, E_rel._ = 0.0 kcal/mol.

**Figure 11:**
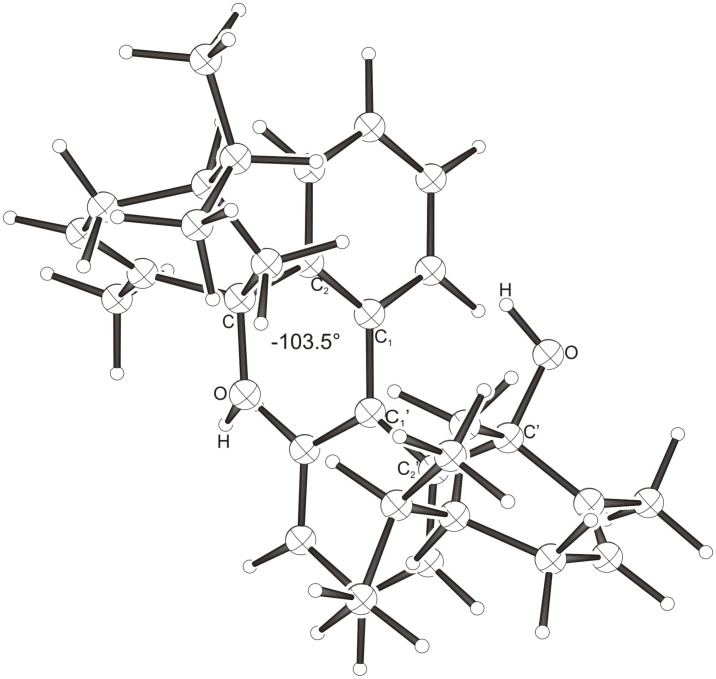
B3LYP/6-31++G**:AM1 optimized structure of **(*M*)-BIMEOL**, E_rel._ = 5.4 kcal/mol.

**Figure 12:**
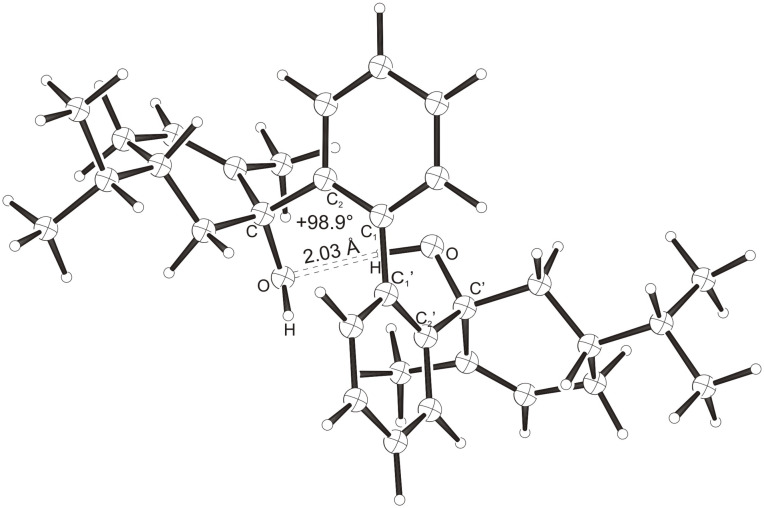
B3LYP/6-31++G**:AM1 optimized structure of **(*P*)-BIMEOL**, E_rel._ = 0.0 kcal/mol.

**Table 3:** Computed absolute and relative energies^a^ of the biphenyl diols in *P* and *M* biphenyl conformations.

Computed structures	ONIOM (B3LYP/6-31++G**: AM1)	E_rel._ [kcal/mol]

**(*M*)-BIMOL**	−152.866360	0.0
**(*P*)-BIMOL**	−152.866159	**+1.3**
**(*M*)-BIVOL**	−152.625276	**+5.1**
**(*P*)-BIVOL**	−152.633361	0.0
**(*M*)-BICOL**	−152.69155	**+5.8**
**(*P*)-BICOL**	−152.70077	0.0
**(*M*)-BIMEOL**	−152.781310	**+5.4**
**(*P*)-BIMEOL**	−152.789859	0.0

^a^The hydroxyl functions were computed with B3LYP/6-31++G**, while AM1 was employed for the rest of the structures. Hydrogen atoms were used as link atoms between the layers.

## Conclusion

The new enantiopure chelating diols **(*M*)-BIMOL**, **(*P*)-BIVOL**, **(*P*)-BICOL** and **(*P*)-BIMEOL** are efficiently accessible from coupling of dilithiobiphenyl with (−)-menthone, (−)-verbenone and (−)-carvone. All diols exhibit flexible biaryl axes, which are however conformationally restricted to *P* or *M* arrangements. These favored biaryl arrangements are apparent from X-ray analyses and computational comparisons of the biphenyl conformers and arise from suitable hydrogen bonding of the chiral terpenol moieties. As it has been demonstrated for the (−)-fenchone derived **(*M*)-BIFOL**, the flexible, but diastereomerically pure, chelating diols can be employed to incorporate many elements or metal ions for the construction of new enantioselective reagents and catalysts.

## Experimental

All reactions were carried out under argon atmosphere using Schlenk tube techniques. Solvents were dried by standard methods and distilled under argon prior to use.

### Synthesis of Biphenyl-2,2′-bismenthol (*M*)-BIMOL

2,2′-Dilithiobiphenyl (2 TMEDA) was synthesized by treating biphenyl with *n*-butyllithium/TMEDA for 24 hours at room temperature [30–31]. To a suspension of 10 g (25.0 mmol) of 2,2′-dilithiobiphenyl (2 TMEDA, M: 398.5 g/mol) in diethyl ether at 0 °C, 7.7 g (8.4 ml, 50.0 mmol) of (−)-menthone were added. The mixture was warmed up to room temperature and was stirred for 24 h. Aqueous work-up followed by 2 recrystallisations from acetone yielded 6.3 g (12.1 mmol, 48%) of **(*M*)-BIMOL**. Analytical and spectroscopic data of **(*M*)-BIMOL**: mp: 182 °C; calcd: C 83.06, H 10.02, found C 82.97, H 10.04; ^1^H NMR (CDCl_3_, 300 MHz) 0.78–0.80 (3H, d), 0.84–0.86 (3H, d), 0.96–0.99 (4H, d), 1.12–1.21 (1H, t), 1.57–1.86 (5H, m), 1.95–2.00 (1H, dd), 2.07–2.12 (1H, d), 2.57 (1H, s), 7.00–7.03 (1H, d), 7.17–7.22 (1H, t), 7.30–7.35 (1H, t), 7.39–7.42 (1H, d); ^13^C NMR (CDCl_3_, 75.5 MHz) 144.77, 132.80, 126.75, 125.54, 125.02, 81.16, 47.90, 35.12, 28.17, 26.57, 24.30, 22.25, 21.65, 18.97; [α]^20^_Na_ = −87.5 (c = 0.4 in *n*-hexane); EI-MS: 462 (M^+^), 426 (M^+^-2H_2_O); IR (NaCl crystal, cm^−1^) 3567 (sharp), 3458 (sharp), 3051 (broad), 2946–2860 (sharp). X-Ray crystal data [32] of **(*M*)-BIMOL **as clathrate with acetone: C_32_H_46_O_2_* C_3_H_6_O, *M *= 520.79 g/mol; space group *P*2_1_2_1_2_1_; *a* = 8.9885(5) Å, *b* = 19.2157(10) Å, *c* = 36.233(2) Å; *V* = 6258.2(6) Å^3^; *Z* = 8; *T* = 100(2) K; μ = 0.068 mm^−1^; reflection total: 31432; unique: 7151; observed: 3612 (I >2σ(I)); parameters refined: 717; R1 = 0.0466, wR2 = 0.0670; GOF = 0.900 (crystallographic data have been deposited with the Cambridge Crystallographic Data Centre).

### Synthesis of Biphenyl-2,2′-bisverbenol (*P*)-BIVOL

To a suspension of 10 g (25.0 mmol) of 2,2′-dilithiobiphenyl (2 TMEDA, M: 398.5g/mol) in diethyl ether at 0 °C, 7.5 g (7.7 ml, 50.0 mmol) of (−)-verbenone were added. The mixture was warmed up to room temperature and was stirred for 24 h. Aqueous work-up followed by 2 recrystallisations from hexane/acetone yielded 4.3 g (6.1 mmol, 24.6 %) of **(*P*)-BIVOL**. Analytical and spectroscopic data of **(*P*)-BIVOL**: mp: 196 °C; calcd: C 81.99, H 8.65, found C 82.19, H 8.64; ^1^H NMR (CDCl_3_, 300 MHz) 0.82 (6H, s), 1.21 (6H, s), 1.31–1.34 (2H, d), 1.66 (2H, s), 2.09–2.20 (4H, m), 2.58 (2H, t), 4.70 (2H, s), 4.76–4.80 (4H, dd), 6.25 (2H, s), 7.23–7.27 (4H, m), 7.31–7.34 (2H, dd), 7.35–7.40 (2H, t); ^13^C NMR (CDCl_3_, 75.5 MHz) 151.04, 139.94, 131.41, 128.38, 127.29, 126.76, 125.09, 107.12, 50.68, 49.16, 43.56, 35.60, 34.46, 26.01, 21.89; [α]^20^_Na_ = −149.3 (*c* = 0.2 in *n*-hexane); EI-MS: 454 (M^+^), 418 (M^+^-2xOH); IR (NaCl plate, cm^−1^) 3237 (sharp), 3053 (broad), 2973–2911 (sharp). X-ray crystal data [32] of **(*P*)-BIVOL **as clathrate with H_2_O: C_32_H_38_O_2_*H_2_O; *M*=472.66 g/mol; space group *P*2_1_3; a = *b* = *c* = 20.029(8) Å; *V* = 8035(6) Å^3^; *Z* = 12; *T* = 100(2) K; μ = 0.072 mm^−1^; reflection total: 27084; unique: 5878; observed: 4143 (I >2σ(I)); parameters refined: 320; R1 = 0.0473, wR2 = 0.0941; GOF = 0.981 (crystallographic data have been deposited with the Cambridge Crystallographic Data Centre).

### Synthesis of Biphenyl-2,2′-biscarvol (*P*)-BICOL

To a suspension of 10 g (25.0 mmol) of 2,2′-dilithiobiphenyl (2 TMEDA, M: 398.5 g/mol) in diethylether at 0 °C, 7.50 g (7.8 ml, 50.0 mmol) of (−)-carvone were added. The mixture was warmed up to room temperature and was stirred for 24 h. Aqueous work-up followed by 2 recrystallisations from hexane/ethyl acetate (10:1) yielded 8.4 g (17.8 mmol, 71%) of **(*P*)-BICOL**. Analytical and spectroscopic data of **(*P*)-BICOL**: mp: 178 °C.; calcd: C 81.32, H 8.53, found C 81.32, H 8.69; ^1^H NMR (CDCl_3_, 300 MHz) 1.61 (6H, s), 1.67 (1H, m), 1.97–1.92 (1H, m), 2.24–2.16 (3H, m), 2.46 (1H, s), 4.64–4.58 (2H, d), 5.72 (1H, s), 7.07–7.04 (2H, m), 7.27–7.24 (2H, dd), 7.33–7.28 (1H, m); ^13^C NMR (CDCl_3_, 75.5 MHz) 148.81, 142.04, 140.75, 136.94, 132.11, 129.18, 126.29, 126.06, 125.40, 108.90, 79.35, 43.99, 37.48, 31.13, 20.73, 17.99; [α]^20^_Na_ = −12.45 (*c* = 0.4 in *n*-hexane); EI-MS: 454 (M^+^), 436 (M^+^-H_2_O); IR (NaCl crystal, cm^−1^) 3391 (sharp), 3058 (broad), 2962–2919 (sharp), 1643. X-ray crystal data [32] of **(*P*)-BICOL **as clathrate with H_2_O: C_32_H_38_O_2_*H_2_O; *M* = 472.66 g/mol; space group *P*2_1_2_1_2_1_; *a* = 12.3983(4) Å, *b* = 12.5961(5) Å, *c* = 17.5876(7) Å; *V* = 2746.7(2) Å^3^; *Z* = 4; *T* = 100(2) K; μ = 0.071 mm^−1^; reflection total: 13337; unique: 3368; observed: 2629 (I >2σ(I)); parameters refined: 380; R1 = 0.0343, wR2 = 0.0659; GOF = 0.960 (crystallographic data have been deposited with the Cambridge Crystallographic Data Centre).

### Synthesis of Biphenyl-2,2′-bis-*p*-menthenol (*P*)-BIMEOL

To a 0 °C cooled solution of 0.5 g of **(*P*)-BICOL **(1.1 mmol) in 30 ml of ethanol a small amount of palladium/C was added. The mixture was placed in an autoclave and was degassed three times with hydrogen. Under 30 bar of H_2_ the mixture was stirred for 24 hours. Then the reaction mixture was filtered, the solvent was removed in vacuo and the resulting white solid was crystallized in ethanol. Recrystallization (two times) from ethanol yielded 0.43 g (0.85 mmol, 77%) of **(*P*)-BIMEOL**. Analytic and spectroscopic data of **(*P*)-BIMEOL**: mp: 204 °C; calcd: C 80.91, H 9.59, found C 80.59, H 9.57; ^1^H NMR (CDCl_3_, 300 MHz) 0.79–0.81 (6H, d), 1.23–1.28 (2H, t), 1.36–1.41 (3H, m), 1.63 (3H, s), 1.75–1.83 (1H, m), 2.09–2.21 (2H, m), 2.55 (1H, s), 3.70–3.77 (1H, q), 5.72 (1H, s), 7.06–7.09 (2H, m), 7.21–7.26 (2H, dd), 7.26–7.33 (1H, m); ^13^C NMR (CDCl_3_, 75.5 MHz) 142.09, 140.87, 137.00, 131.98, 129.34, 126.16, 125.85, 125.79, 79.45, 43.26, 36.43, 32.11, 29.17, 19.85, 19.23, 17.92; [α]^20^_Na_ = −51.7 (*c* = 0.4 in *n*-hexane); EI-MS: 458 (M^+^), 440 (M^+^-H_2_O); IR (NaCl crystal, cm^−1^) 3349 (sharp), 3046 (broad), 2957-2880 (sharp); X-ray crystal data [32] of **(*P*)-BIMEOL** as clathrate with ethanol: C_32_H_42_O_2_*C_2_H_6_O; *M*=504.74 g/mol; space group *P*2_1_2_1_2_1_; *a* = 11.4105(1) Å, *b* = 11.9179(2) Å, *c* = 12.3467(3) Å; *V* = 3038.91(7) Å^3^; *Z* = 4; *T* = 200(2) K; μ = 0.068 mm^−1^; reflection total: 25469; unique: 3720; observed: 3028 (I >2σ(I)); parameters refined: 441; R1 = 0.0410, wR2 = 0.1097; GOF = 1.180 (crystallographic data have been deposited with the Cambridge Crystallographic Data Centre).

## Computational Section

All computed structures were fully optimized using Morokuma's ONIOM method implemented in GAUSSIAN98 [33]. Hybrid DFT (B3LYP/6-31++G*) levels of theory were applied to the hydroxyl groups, while the rest of the structures were computed by the semiempirical AM1 method. Hydrogen atoms were used as link atoms between the two layers. The structures were analyzed by frequency computations and showed no imaginary frequencies.
